# Supplementary material for: Rate-selected growth of ultrapure semiconducting carbon nanotube arrays
Source: Nat Commun. 2019 Oct 2;10:4467. doi: 10.1038/s41467-019-12519-5 (PMC6775125; doi:10.1038/s41467-019-12519-5)
Supplement: Supplementary file 1 — Supplementary Information [file 41467_2019_12519_MOESM1_ESM.pdf]

Supplementary Materials for

Rate-selected growth of ultrapure semiconducting carbon nanotube arrays

Zhu et al.

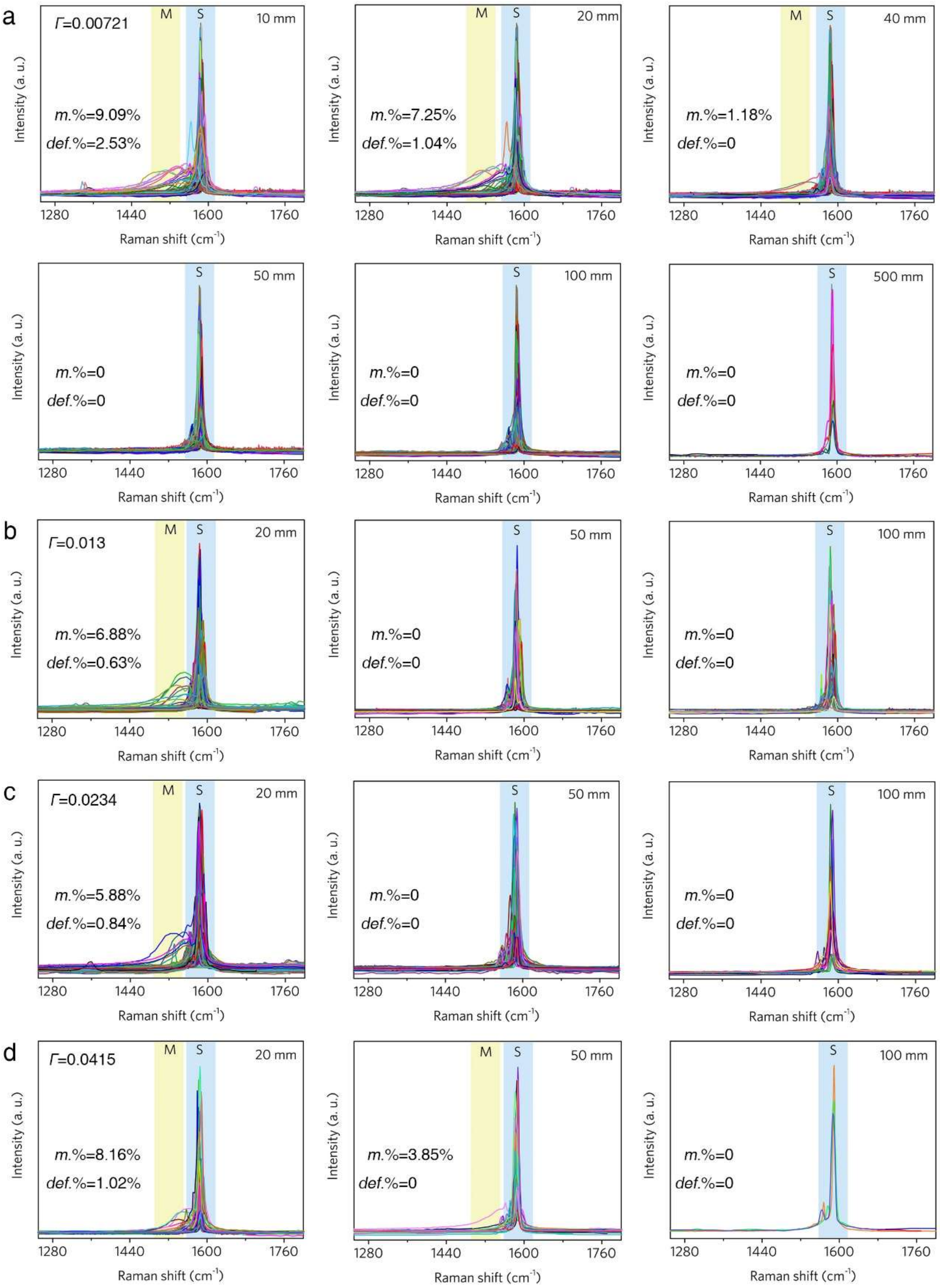

Supplementary Figure 1 | Raman spectra at various length of aligned CNTs grown at different conditions. a, *T*=1010 ℃, w_{H2O}=0.45%; **b,** *T*=1010 ℃, w_{H2O}=0.55%; **c,** *T*=980 ℃, w_{H2O}=0.45%; **d,** *T*=980 ℃, w_{H2O}=0.55%. *m.*% represents the m-CNT percentage of aligned tubes at specific length while *def.*% denotes the ratio of defective CNTs.

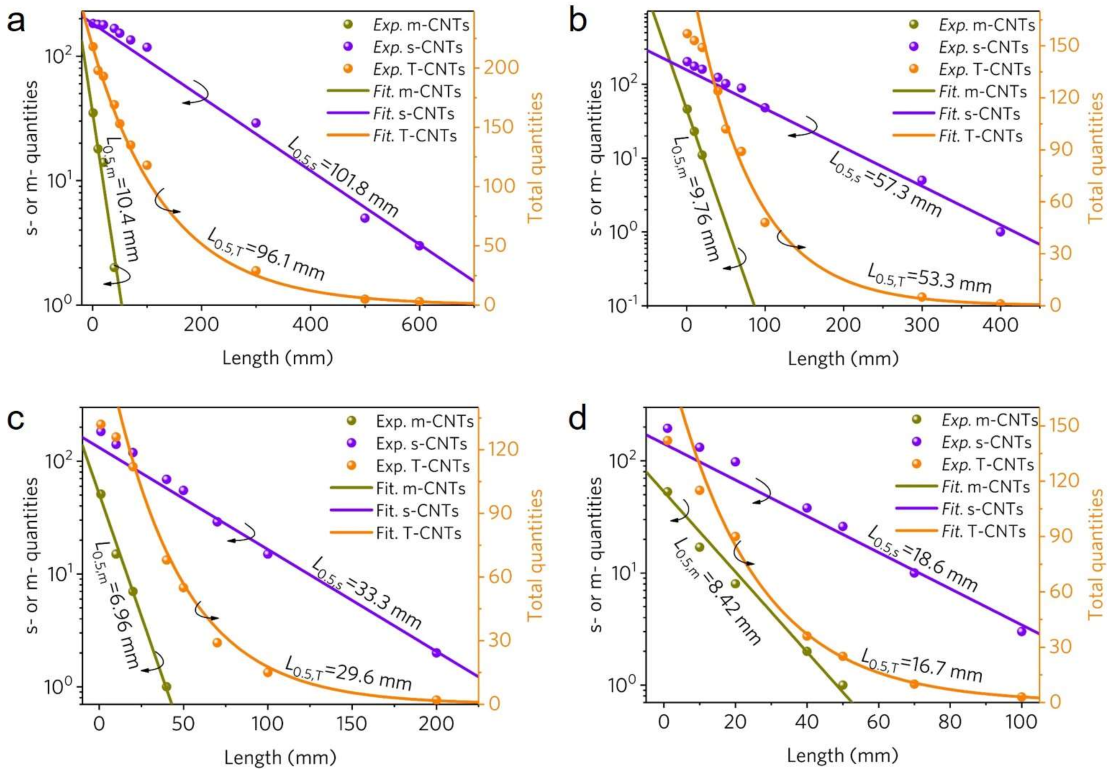

Supplementary Figure 2 | Exponential decay in quantities of m-, s-, and total CNTs with length. The CNT quantities were statistically counted based on the corresponding samples shown in Supplementary Figure 1. The s- and m-CNT quantities correspond to the left axis in logarithmic scale while the total CNT quantities correspond to the right axis in normal scale. $L_{0.5,s}$, $L_{0.5,m}$ and $L_{0.5,T}$ represent the half length of s-CNTs, m-CNTs and total CNTs, respectively.

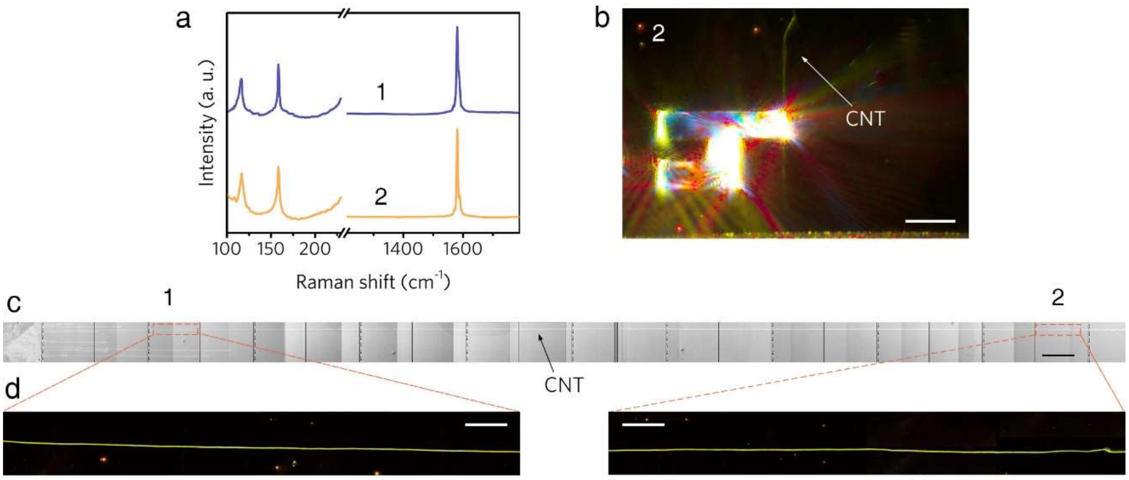

Supplementary Figure 3 | Chiral consistency of an individual DWCNT longer than 35 mm. The morphology of the DWCNT was characterized by SEM **c**, with its head and tail visualized in uniform color under resonant Rayleigh scattering **b, d**. Also, the head and tail part of the DWCNT possessed the same Raman peak positions without structural defects **a**, further indicating the chiral consistency and structural perfectness of the DWCNT. Scale bars, 20 μm in **b**, **d** and 1 mm in **c**.

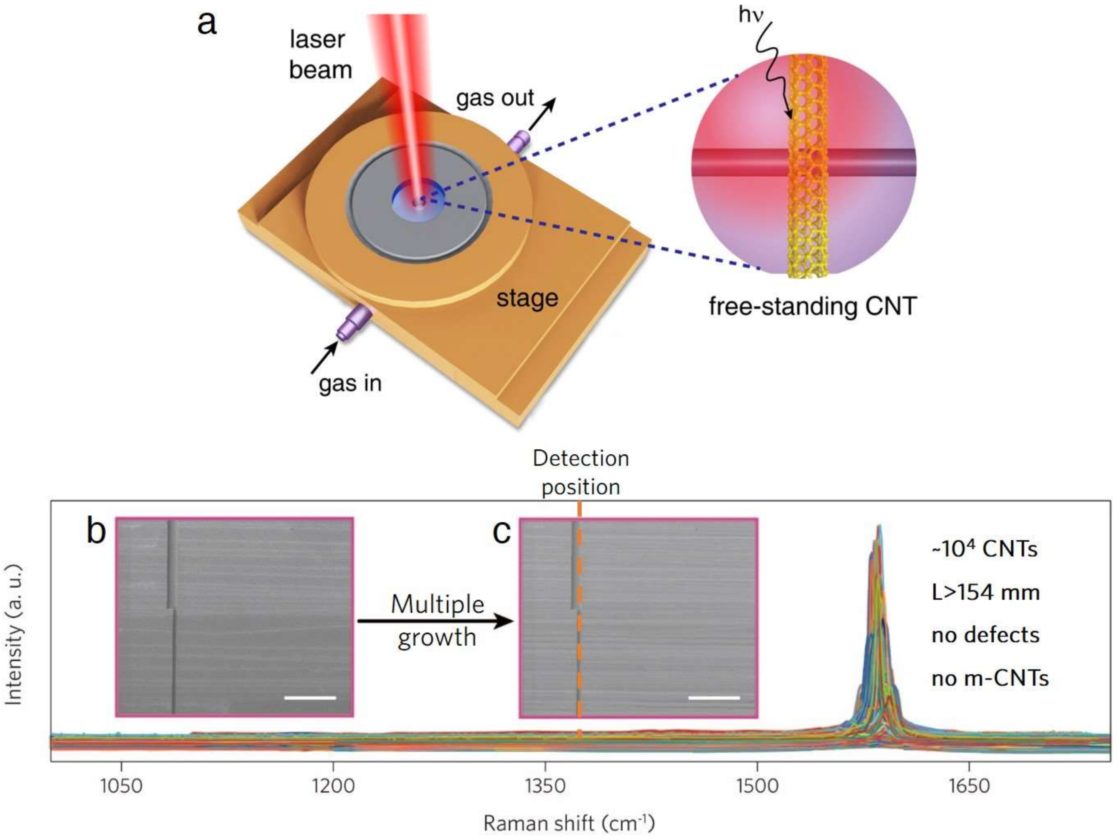

Supplementary Figure 4 | Micro-Raman measurements of ~10^4 CNTs longer than 154 mm. The aligned CNTs **b** longer than 154 mm were synthesized at condition T=1020 ℃, w_{H2O}=0.5%, V_{H2}: V_{CH4} = 2:1, followed by ten-time multiple growth **c** with the preloaded catalysts. Preceded the sequential micro-Raman measurements on individual CNTs, the as-grown samples encapsulated in a stage **a**, went through an annealing treatment at 450 ℃ under argon atmosphere, in case of a smear-out on the BWF characteristics caused by O_2-adsorption-induced Fermi level shifts. These suspended tubes after annealed treatment, which were statistically counted one-by-one, can best exhibit their pristine characteristics with a notably enhanced intensity. Scale bars in **b**, **c** are 300 μm.

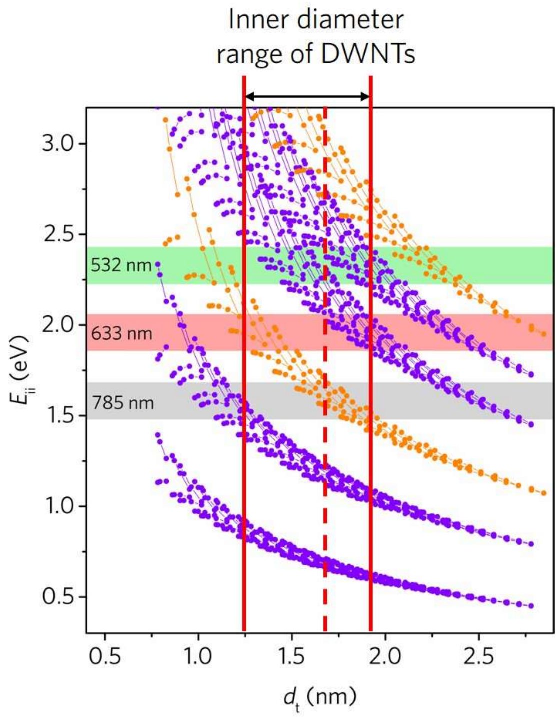

Supplementary Figure 5 | Resonance of the MWNTs with laser irradiation wavelength. Normalized Kataura plot: orange (purple) symbols, transition energies for metallic (semiconducting) SWNTs; Rectangular bars, laser excitation energies used in this work; Vertical lines: inner diameter range of DWNTs; Dotted line: predominated inner diameter. Outer diameters of DWNTs distribute in the range of 2.0~3.5 nm while inner ones range from 1.30 to 1.80 nm, which center around 1.7 nm. This corresponds to the E_{ii} ranges of 1.5~2.0 eV and 2.5~3.0 eV in the Kataura plot regarding the resonance of metallic components. However, the m-CNTs in the E_{ii} range of 2.5~3.0 eV are mostly families with high indexes (2*n*+*m*=36, 39, 42), which are seldom found in our as-grown CNTs. Mono-wavelength lasers (2.33 eV, 1.96 eV, 1.58 eV) have covered most of the common tube families including the major distribution of m-CNTs. Resonance of at least one layer with the laser determines the excitation of both the concentric layers due to quantum coupling.

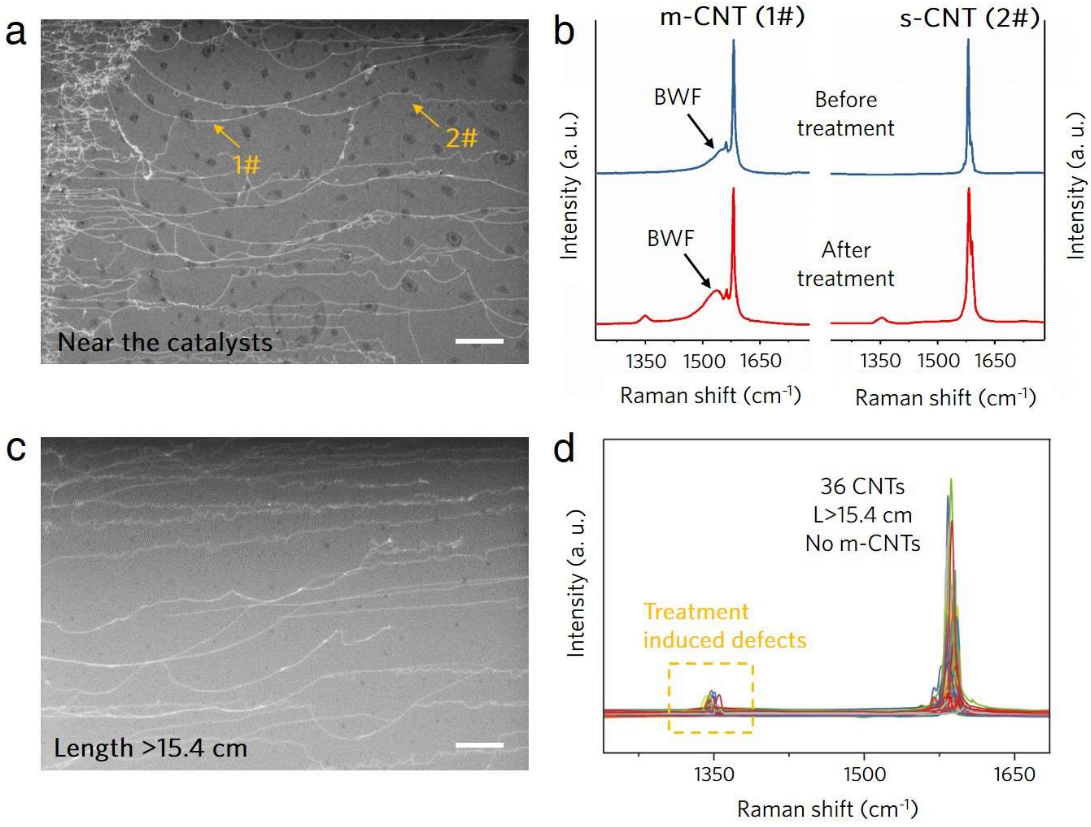

Supplementary Figure 6 | SEM images of the CNTs near the catalysts **a** and those longer than 154 mm **c** after surfactant treatment. Scale bars, 100 μm for **a** and **c**. **b**, Raman G modes of two typical CNTs near the catalysts before and after surfactant treatment. **d**, A collective of G modes for 36 CNTs longer than 154 mm. An obvious enhancement of BWF intensity after surfactant treatment for a same m-CNT (1# in **b**) validated the efficiency of this method. Therefore, no detection of BWF lineshapes for tubes longer than 154 mm after surfactant treatment verified further the high selectivity of s-CNTs.

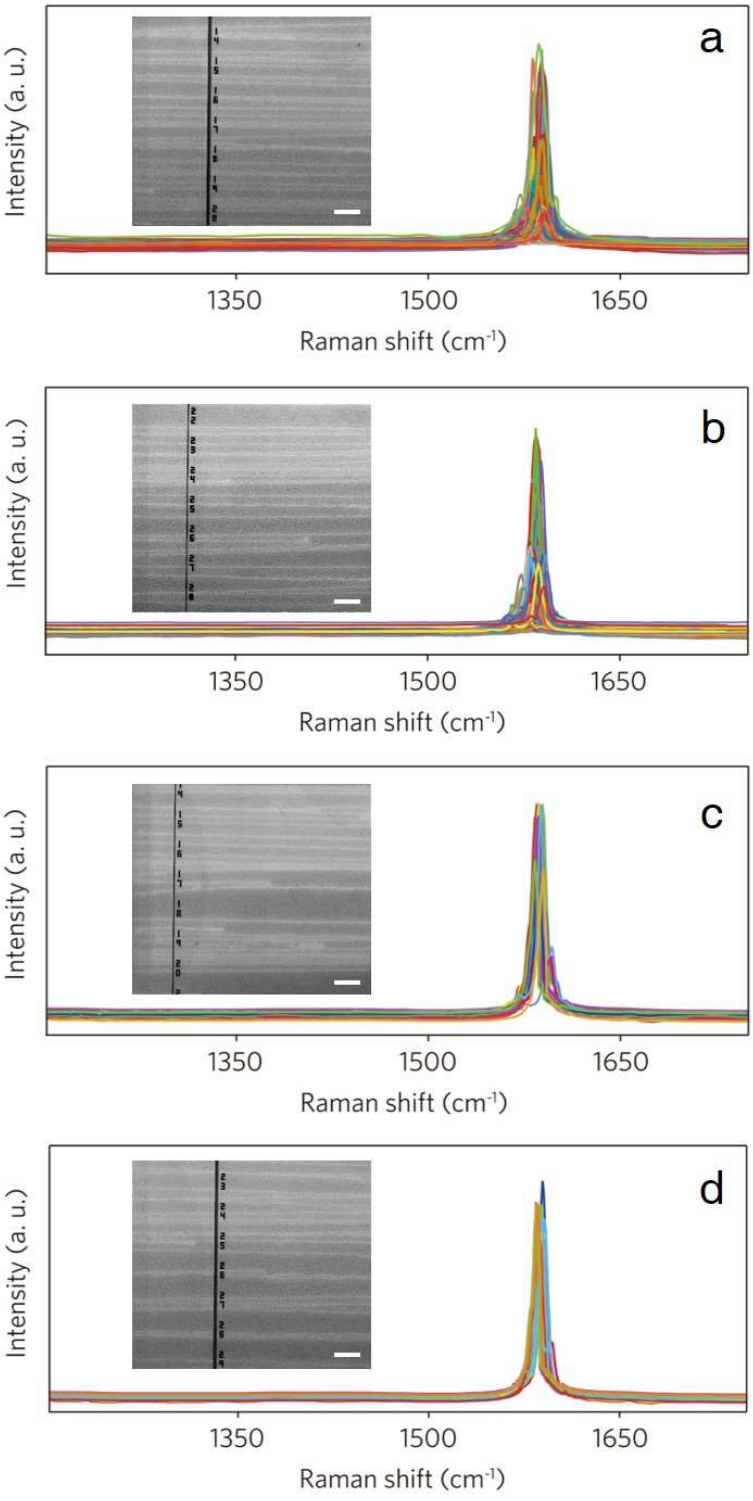

Supplementary Figure 7 | Raman spectra of CNTs longer than 154 mm synthesized at different conditions. a, *T*=1010 ℃, w_{H2O}=0.4%; **b,** *T*=1000 ℃, w_{H2O}=0.4%; **c,** *T*=1010 ℃, w_{H2O}=0.5%; **d,** *T*=1000 ℃, w_{H2O}=0.5%. Scale bars in **a-d** are 200 μm.

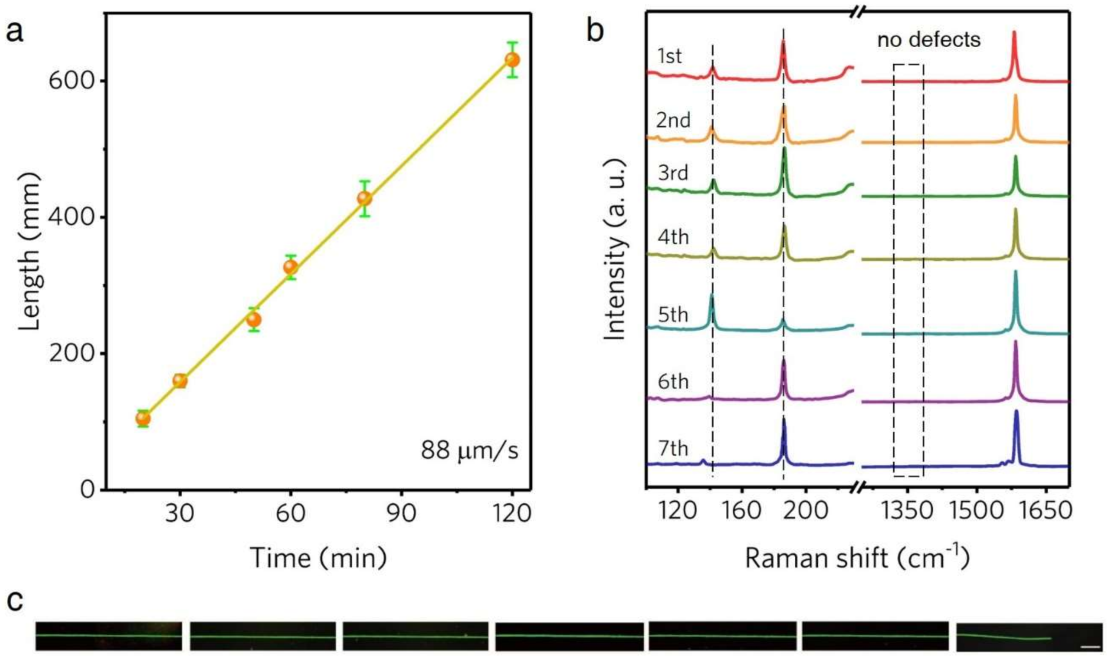

Supplementary Figure 8 | Characterization of the 650-mm-long CNT. a, Dependence of the average CNT length on their growth time. For CNTs with different length on substrates, only the longest 10 CNTs were taken into consideration for calculating the growth rate. Error bars represent standard deviation of the length for tubes grown at different growth time. **b,** RBM mode waterfall of the longest CNT. Each spectrum was probed from the seven different wafers along the long tube. **c,** Rayleigh images of the 650-mm-long CNT captured from each wafer. Scale bar, 200 μm.

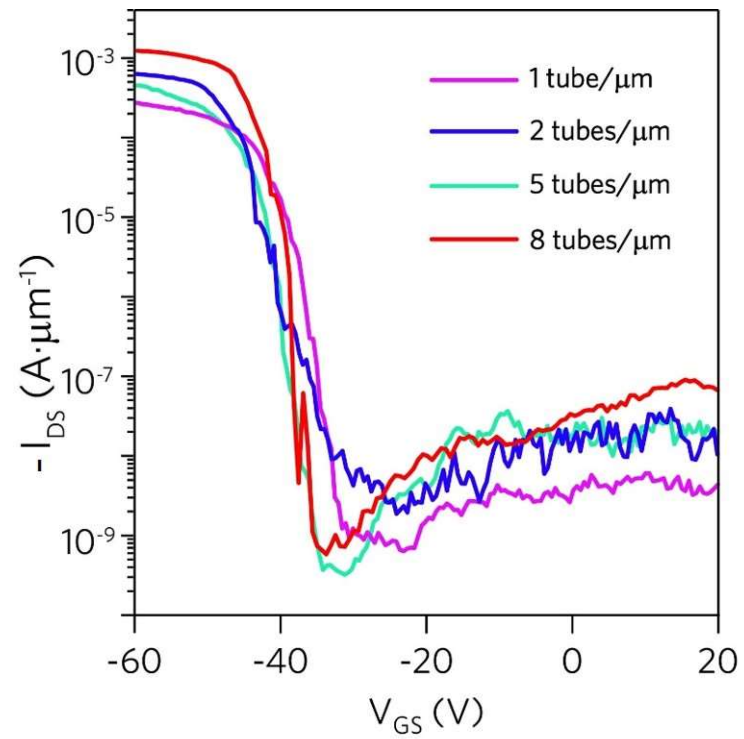

Supplementary Figure 9 | Transfer characteristics of transistors fabricated on CNTs longer than 154 mm after each growth cycle. The devices were built with interdigitated electrodes driven by a same applied V_{DS} of -0.1 V.

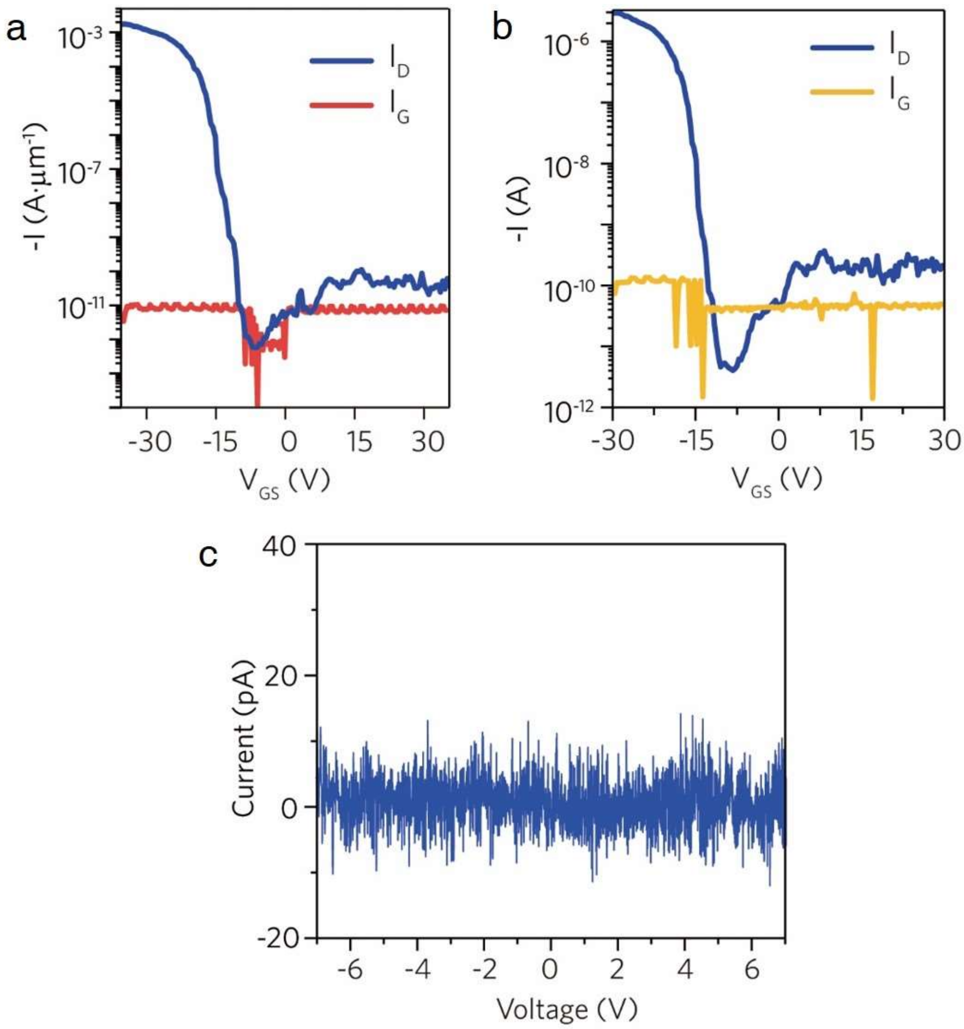

Supplementary Figure 10 | **a**, the gate current I_G versus V_{GS} of the device shown in Fig. 2h where I_{DS} is also measured as a function of V_{GS}. The current is width-normalized with applied V_{DS} of -0.1 V. I_G couldn't be the result of leakage from the gate. Because it would behave as a resistor conforming to the Ohm's Law when there is current leakage, instead of retaining the positive current direction during the voltage sweeping. We suppose that it might be caused by the capacitance, of which the absolute value depends on multiple factors like scanning rate, scanning direction, etc. The lower I_G near the current valley can be attributed to this capacitance effect as well, given the lower scan rate (longer integration time) in measuring the ultralow current. I_G have a limited impact on the on/off ratio due to its similar values to the stable off current. The areal off current can be estimated as 'measured off current±gate current', which still enables the on/off ratio higher than 10^8. **b,** Transfer characteristic of a transistor fabricated on one ultralong CNT plotted in logarithmic scale with applied V_{DS} of -0.5 V. The corresponding I_G versus V_{GS} data is also shown in yellow. **c**, Transfer characteristic measured in transistors without CNTs.

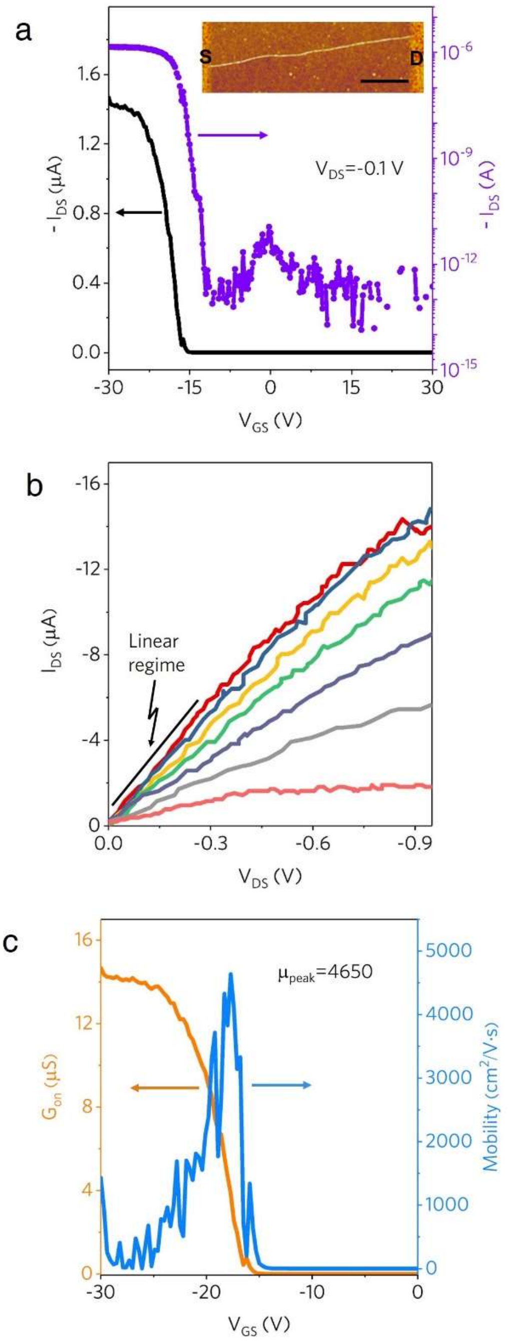

Supplementary Figure 11 | Electrical performances of a representative transistor. a, Transfer characteristics of a typical transistor fabricated on a CNT longer than 154 mm with an applied voltage of V_{DS}=-0.1 V, plotted in both linear (lines, left axis) and logarithmic (symbol lines, right axis) scales. Inset, AFM topography of the transistor device structure. Scale bar, 1 μm. **b,** Output characteristic of the transistor measured with ascending V_{GS} from -11 V to -17 V at a step of -1 V. **c,** The relationship of on-state conductance and mobility versus V_{GS} as derived from the transfer characteristics.

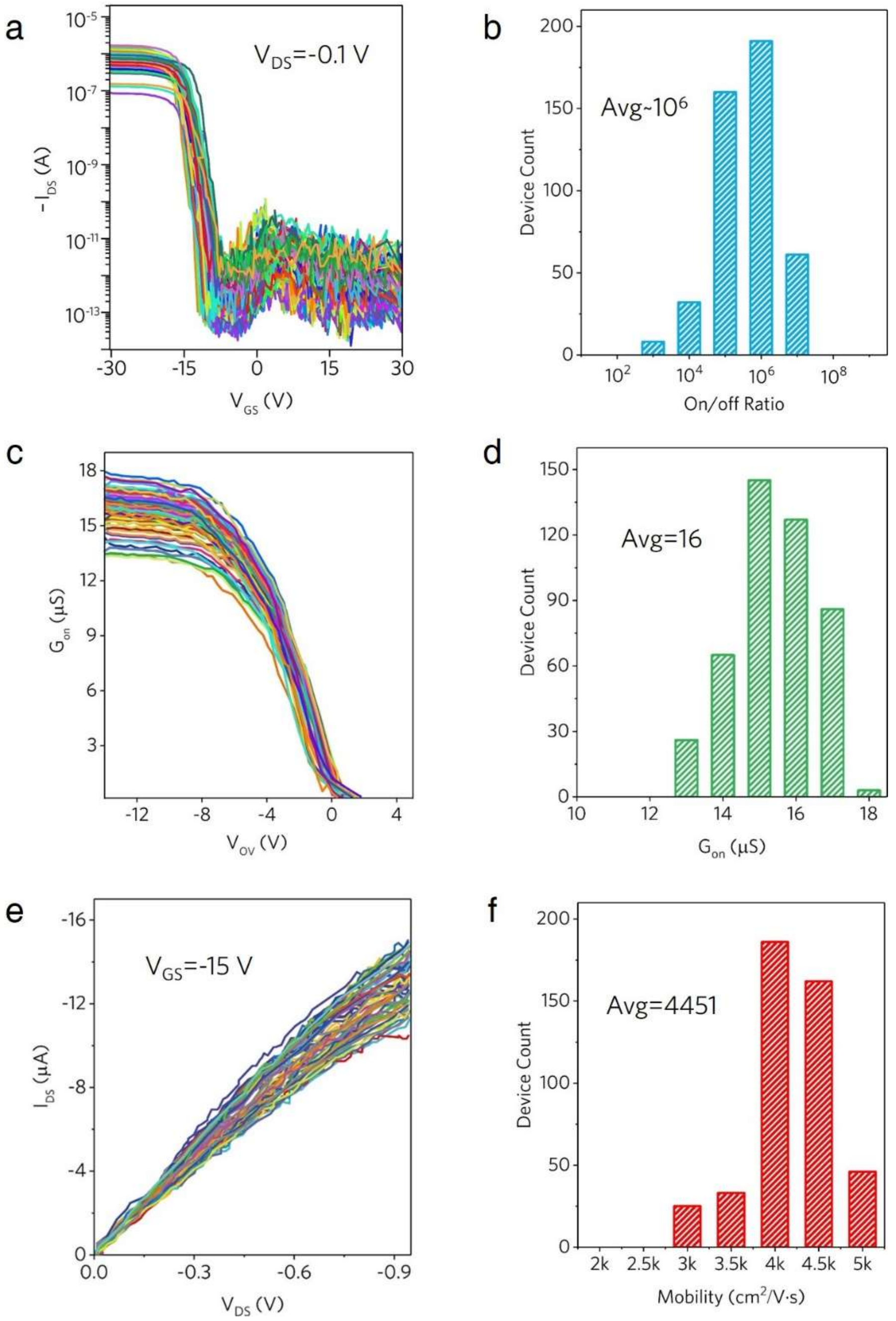

Supplementary Figure 12 | Statistics of the electrical performances of single-tube transistors. a, Collection of transfer curves measured for 452 transistors each built on an individual CNT with length exceeding 154 mm. **b,** The histogram showing the distribution of on/off ratio as measured in **a**. **c,** Collection of on-state conductance curves extracted from **a**, V_{OV} denotes how much the gate voltage was applied above the threshold voltage. **d,** The histogram showing the distribution of on-state conductance as measured in **c**. **e,** Collection of output characteristics with an applied V_{GS} of -15 V. **f,** The histogram showing the distribution of mobility extracted from **a**. Avg. in **b**, **d**, **f** represents the average value.

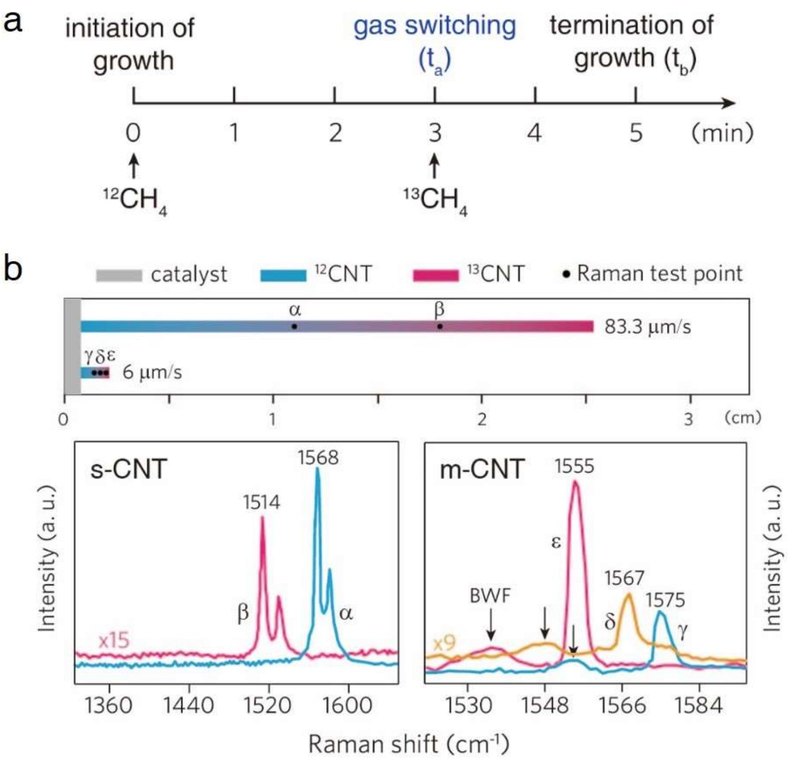

Supplementary Figure 13 | a, Graphic diagram for the isotope switching test. For the first 3 min, the CNTs were synthesized from cracked $^{12}CH_4$, preceded the synthesis with $^{13}CH_4$ as the feedstock for the last 2 min. The feedstock was switched to $^{13}CH_4$ at t_a =3 min and the reaction was terminated at t_b =5 min. **b**, Schematic of growth rate measurement with the isotope switching method. The bottom panels are G modes of typical s-CNT and m-CNT before and after isotope switching. This shows that an individual s-CNT possess higher growth rate than that of m-CNT.

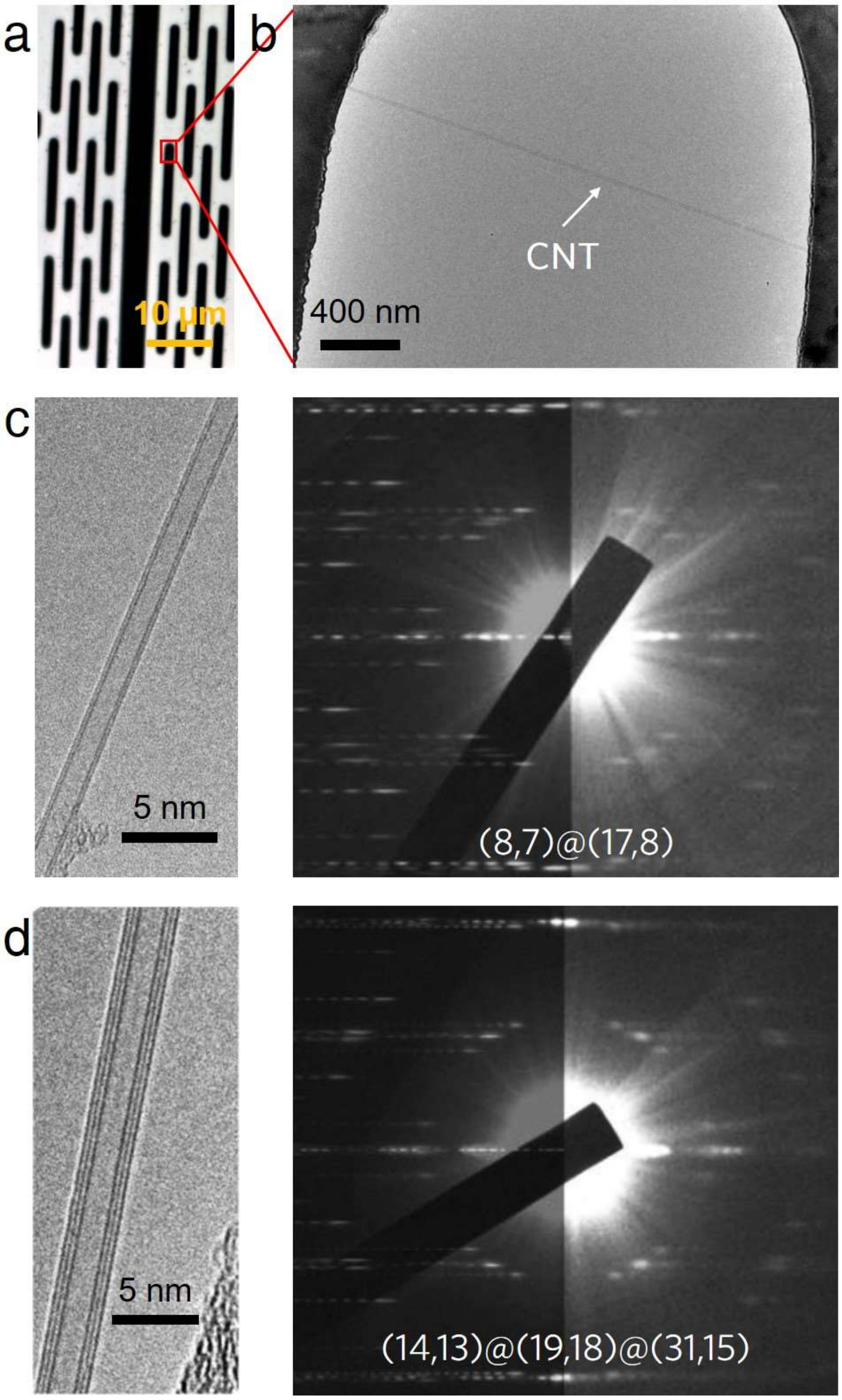

Supplementary Figure 14 | Assignment of chiral indexes by electron diffraction for ultralong CNTs. a, Optical image of the trenched Si/SiO_2 grid. **b,** Expanded view of the grid as in (a) under TEM. **c, d,** TEM images and the corresponding ED patterns of the representative free-standing m-CNT indexed to (8,7)@(17,8) and s-CNT indexed to (14,13)@(19,18)@(31,15).

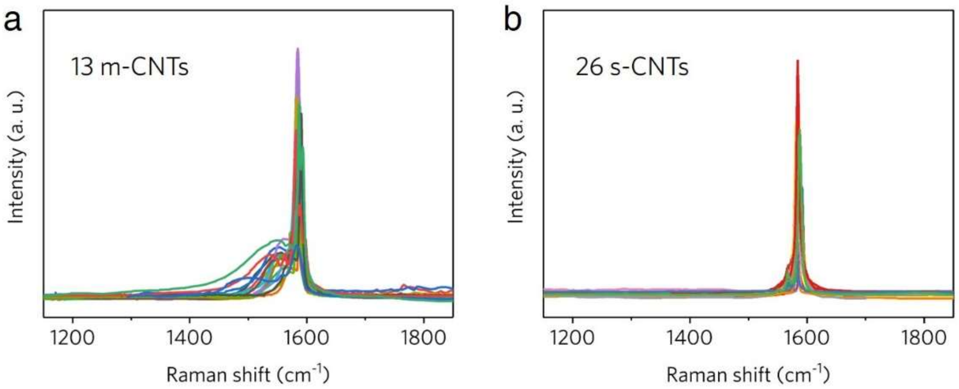

Supplementary Figure 15 | Raman spectra of the m-^{12}CNTs (**a**) and s-^{12}CNTs (**b**) corresponding to those characterized under TEM.

Supplementary Table 1 | TOF versus bandgap for s-CNTs

CNT type	TOF (s^{-1})	n	m	d (nm)	θ (°)	E (eV)
TWNTs	1320345	14	13	1.83	28.8	0.45
		24	14	2.61	21.4	0.32
		29	19	3.28	23.1	0.25
	1583951	11	9	1.36	26.7	0.61
		19	12	2.12	22.6	0.39
		26	16	2.87	22.2	0.29
	1354651	15	14	1.97	28.9	0.42
		20	19	2.65	29.2	0.31
		32	16	3.31	19.1	0.25
	1213867	14	13	1.83	28.8	0.45
		19	18	2.51	29.1	0.33
		31	15	3.18	18.6	0.26
	1036429	21	19	2.71	28.3	0.30
		30	23	3.60	25.6	0.23
		33	31	4.34	29.0	0.19
	988830	30	20	3.41	23.4	0.24
		44	19	4.38	17.1	0.19
		48	25	5.03	19.7	0.16
DWNTs	301901	9	7	1.09	25.9	0.76
		14	12	1.76	27.5	0.47
	184984	10	5	1.04	19.1	0.80
		14	12	1.76	27.5	0.47
	960300	19	12	2.12	22.6	0.39
		9	7	1.09	25.9	0.76
	194296	13	12	1.70	28.7	0.49
		8	7	1.02	27.8	0.81
	260735	13	6	1.32	18.0	0.63
		19	8	1.88	16.8	0.44
	837482	13	8	1.44	22.2	0.57
		21	7	1.98	13.9	0.42
	1675470	13	9	1.50	24.0	0.55
		24	7	2.21	12.4	0.37
	1320073	22	11	2.28	19.1	0.36
		27	17	3.01	22.5	0.27
	951086	12	8	1.37	23.4	0.60
		16	14	2.04	27.8	0.40
	1634996	16	12	1.91	25.3	0.43
		27	10	2.60	15.1	0.32
	1386630	10	9	1.29	28.3	0.64
		18	11	1.99	22.1	0.41
	1490634	20	4	1.74	8.95	0.47
		20	16	2.45	26.3	0.34

	954797	13	9	1.50	24.0	0.55
		24	7	2.21	12.4	0.37
	1398959	22	11	2.28	19.1	0.36
		27	17	3.01	22.5	0.27
	1641445	27	5	2.33	8.35	0.35
		18	5	1.64	11.9	0.50
	1764944	18	5	1.64	11.9	0.50
		27	5	2.33	8.35	0.35
	1487639	24	1	1.92	2.02	0.43
		20	19	2.65	29.2	0.31
	1809276	19	3	1.62	7.22	0.51
		19	15	2.31	26.1	0.36
	1583391	14	12	1.76	27.5	0.47
		23	13	2.47	20.9	0.33
	1486939	18	5	1.64	11.9	0.50
		27	5	2.33	8.35	0.35

Supplementary Table 2 | TOF versus bandgap for m-CNTs

CNT type	TOF (s^{-1})	n	m	d (nm)	θ (°)	E (eV)
TWNTs	55741	32	18	3.43	20.8	0.24
		43	19	4.31	17.4	0.00047
		50	24	5.12	18.5	0.16
	105668	18	5	1.64	11.9	0.50
		24	8	2.26	13.9	0.36
		35	8	3.10	10.1	0.00091
	44550	35	20	3.78	21.1	0.00061
		45	19	4.46	16.8	0.18
		51	24	5.19	18.3	0.00032
	201237	49	6	4.09	5.71	0.00052
		33	13	3.22	15.9	0.26
		20	14	2.32	24.2	0.36
DWNTs	487350	8	7	1.02	27.8	0.81
		17	8	1.73	18.3	0.0031
	245438	15	12	1.83	26.3	0.0027
		8	6	0.95	25.3	0.86
	433125	15	10	1.71	23.4	0.48
		27	6	2.38	9.83	0.0015
	149725	20	9	2.01	17.6	0.41
		27	12	2.71	17.5	0.0012
	72877	27	2	2.20	3.54	0.37
		26	17	2.94	23.1	0.0010
	324736	23	10	2.29	17.2	0.36
		32	11	3.03	14.3	0.00095
	362070	15	13	1.90	27.6	0.43
		31	4	2.60	5.99	0.0013
	2494800	22	14	2.46	22.7	0.33
		40	1	3.17	1.22	0.00087
	372600	31	4	2.60	5.99	0.0013
		15	13	1.90	27.6	0.43

Note: The values in red in Supplementary Tables 1-2 represent the minimum bandgap for the corresponding few-walled CNTs.
